# Genome Sequences and Characterization of Chicken Astrovirus and Avian Nephritis Virus from Tanzanian Live Bird Markets

**DOI:** 10.3390/v15061247

**Published:** 2023-05-25

**Authors:** Henry M. Kariithi, Jeremy D. Volkening, Gaspar H. Chiwanga, Mary J. Pantin-Jackwood, Peter L. M. Msoffe, David L. Suarez

**Affiliations:** 1 Exotic and Emerging Avian Viral Diseases Research Unit, Southeast Poultry Research Laboratory, U.S. National Poultry Research Center, Agricultural Research Service, USDA, Athens, GA 30605, USA; 2 Biotechnology Research Institute, Kenya Agricultural and Livestock Research Organization, Kaptagat Rd, Nairobi P.O. Box 57811-00200, Kenya; 3 BASE2BIO, Oshkosh, WI 54904, USA; 4 Tanzania Veterinary Laboratory Agency, South Zone, Mtwara P.O. Box 186, Tanzania; 5 Department of Veterinary Medicine and Public Health, Sokoine University of Agriculture, Chuo Kikuu, Morogoro P.O. Box 3000, Tanzania

**Keywords:** *Astroviridae*, AAstV, ANV, CAstV, de novo assembly, genogroup, NGS, recombination

## Abstract

The enteric chicken astrovirus (CAstV) and avian nephritis virus (ANV) are the type species of the genus *Avastrovirus* (AAstV; *Astroviridae* family), capable of causing considerable production losses in poultry. Using next-generation sequencing of a cloacal swab from a backyard chicken in Tanzania, we assembled genome sequences of ANV and CAstV (6918 nt and 7318 nt in length, respectively, excluding poly(A) tails, which have a typical AAstV genome architecture (5′-UTR-ORF1a-ORF1b-ORF2-‘3-UTR). They are most similar to strains ck/ANV/BR/RS/6R/15 (82.72%) and ck/CAstV/PL/G059/14 (82.23%), respectively. Phylogenetic and sequence analyses of the genomes and the three open reading frames (ORFs) grouped the Tanzanian ANV and CAstV strains with Eurasian ANV-5 and CAstV-Aii viruses, respectively. Compared to other AAstVs, the Tanzanian strains have numerous amino acid variations (substitutions, insertions and deletions) in the spike region of the capsid protein. Furthermore, CAstV-A has a 4018 nt recombinant fragment in the ORF1a/1b genomic region, predicted to be from Eurasian CAstV-Bi and Bvi parental strains. These data should inform future epidemiological studies and options for AAstV diagnostics and vaccines.

## 1. Introduction

Chicken astrovirus (CAstV) and avian nephritis virus (ANV) of the genus *Avastrovirus* (AAstV; *Astroviridae* family [1]) are emerging poultry pathogens capable of causing significant production losses in commercial poultry [2,3,4]. Their infections are usually asymptomatic in adult domestic birds, but in young birds they can cause enteritis (in turkeys, chickens and guineafowls), nephritis (in chickens) and hepatitis (in ducks and geese), and induce runting-stunting syndrome (RSS), white-chick syndrome (WCS) and immunosuppression in various species [5,6,7]. Multiple AAstVs as well as other enteric viruses, such as avian adenoviruses, rotaviruses, reoviruses and coronaviruses, can coinfect poultry [8,9,10].

The infectious polyadenylated, positive-sense ssRNA linear molecule of the AAstVs (6.4–7.9 kb in size) serves as both the viral genome and mRNA, and contains three open reading frames (ORF1a, ORF1b and ORF2) flanked by 5′-and 3′-unstranslated regions (UTRs). ORF1a encodes three viral non-structural polypeptides (nsP)—a 3C-like serine protease, a viroporin, a nuclear localization signal (NLS), and in some cases, a viral genome-linked protein (VPg) [1,11,12]. ORF1b, the most conserved genomic region, encodes the viral RNA-dependent RNA polymerase (RdRp), and ORF2 encodes the VP90 capsid protein (CP) precursor. The cleavage of VP90 by host caspases produces VP70, which after post-translational cleavage by extracellular trypsin proteases produces the structural peptides VP34 (inner core; S), VP27 (outer core; P1) and VP25 (spike; P2). The largely conserved core (S-P1) domain interacts with the viral genome, while P2, which forms the signature 5-/6-star-like surface spike features of AAstVs, is the most variable region and interacts with the host’s cell receptors and immune system [11,12].

The AAstV classification has been amended several times since the mid-70s and serotyping remains unresolved largely due to difficulties in their isolation in cell cultures, which also has hindered cost-effective vaccine production [5,13,14]. Because the classification does not necessarily correspond to the phylogeny, group members are recognized based on the genetic distances of the complete CP amino acid (aa) sequences, where genotypes are separated by genetic distances of 0.576–0.741 and the species within the genotypes are separated by genetic distances of 0.204–0.284 [15,16,17,18]. Current knowledge of AAstVs is biased against wild avian species because the majority of the available genomic data are in domestic poultry viruses; some AAstV strains remain uncharacterized and unassigned [19]. Currently, three AAstV groups are recognized: AAstV-I (turkey astrovirus types 1 and 2 (TAstV 1–2)), AAstV-II (avian nephritis virus types 1–3 (ANV 1–3) and chicken astroviruses (CAstVs)), and AAstV-III (duck astrovirus types 1 and 2 (DAstV 1–2)) [1,20,21]. Other AAstVs have been reported in geese (GoAstV 1–2; [22,23,24]), guinea fowl (GfAstV; [25,26]), pigeons (PiAstV; [16]), passerines (PasAstV; [6]) and other avian species [27]. Based on the CP sequences, CAstVs are further divided into antigenic groups A and B with subgroups Ai–Aiii and Bi–Bvi, respectively [20,28,29,30,31,32]. Furthermore, the emergence of variants has expanded the ANVs from 3 (ANV 1–3) to 11 groups based on the ICTV’s criterion of genetic distances of the CP sequences [21,33,34]. Despite their general host-specificity, intra-/cross-species transmission of AAstVs is not uncommon [12,35].

Although they are globally infecting diverse avian species, the prevalence and epidemiology of AAstVs in the African poultry industry remain largely unknown. The vast majority of currently available AAstV genomic data are from Sanger sequencing of polymerase chain reaction amplicons. Using nontargeted next-generation sequencing (NGS) of a cloacal sample from an adult backyard chicken from Tanzania, we assembled and molecularly characterized the genome sequences of the ANV and CAstV strains.

## 2. Materials and Methods

### 2.1. Samples, RNA Extraction and NGS

The oropharyngeal (OP) and cloacal (CL) samples used in the current study were part of a consignment of samples from backyard chickens collected at live bird markets (LBMs) in Arusha, Dar es Salaam, Iringa, Mbeya, Morogoro and Tanga in Tanzania, during surveillance of the Newcastle disease virus (NDV) conducted between September 2018 and May 2019. From each bird, one OP and one CL was collected. During the sampling period, the flocks did not present overt clinical signs consistent with avian diseases, and their vaccination statuses or histories were not available. After collection using standard procedures [36], the samples were shipped to the Southeast Poultry Research Laboratory (SEPRL) in Athens, GA, USA for total RNA extraction using the MagMAX™-96 AI/ND Viral RNA Isolation Kit (Thermo Fisher Scientific, Waltham, MA, USA) as recently described [37]. Preparation of sequencing libraries (sequence-independent, single-primer amplification [38] and Nextera ^TM^ Flex protocols) and paired-end NGS (500-cycle MiSeq Reagent Kit v3) using the Illumina MiSeq platform were performed as previously described [39]. For the current study, 20 birds that were NDV-positive by real-time reverse transcription–polymerase chain reaction (rRT–PCR) were selected (n = 40 samples; one OP and one CL from each of the 20 birds).

### 2.2. Genome Sequence Assembly and Characterization

Raw NGS data were processed using a nontargeted classification and *de novo* assembly pipeline developed by BASE₂BIO LLC (Oshkosh, WI, USA). Specifically, for the consensus genome sequences published here, raw reads were pre-processed using Trim Galore v0.6.7 (github.com/FelixKrueger/TrimGalore) to remove residual sequencing adapters, SISPA primers, and very-low-quality 3′ ends (q < 8). Host reads were removed using a BBTools (https://sourceforge.net/projects/bbmap/, accessed on February 2023) bbduk filter against the chicken genome build bGalGal1.mat.broiler.GRCg7b, with k = 35, hdist = 0, mincovfraction = 0.3. Final assembly was performed with MEGAHIT v1.2.9 [40] with default parameters. Assemblies were quality-reviewed by mapping trimmed reads against the assembly using BWA-MEM [41] with default parameters and inspecting them with an integrative genomics viewer [42] for obvious coverage or assembly artifacts. The assembled consensus sequences were annotated using Geneious Prime^®^ v2023.1.1 (www.geneious.com, accessed on 10 March 2023) as recently described [43,44]. Sequences from this study and other AAstVs (retrieved from GenBank) were aligned using MAFFT v7.490 [45], trimmed using trimAl v1.2 [46] and used for phylogenetic analysis using the Maximum Likelihood method in MEGA 11 (1000 bootstrap replicates) [47]. Recombination events were assessed using Recombination Detection Program 4 (RDP4) v4.101 as described previously [44,48,49].

## 3. Results

### 3.1. Nontargeted Virus Discovery and Genome Assembly

The NGS detected AAstV-specific RNAs in a CL sample from a chicken (ID IM162) sampled in April 2019 from Miomboni LBM located in the urban district of Iringa, Tanzania; the counterpart OP swab did not contain detectable AAstV RNAs. The CL sample contained 15,643 and 9393 reads specific to ANV and CAstV (based on BLASTn), respectively, which were assembled *de novo* into full-length genome sequences of ANV and CAstV strains (size of 6919 and 7318 nt, excluding the poly(A)tail; single contigs with median read depth coverage of 1502X and 479X, respectively). In addition to AAstVs, genetic sequences of bacterial and viral species of avian interest were identified, including *Enterococcus* sp. (*E. cecorum* and *E. faecium*) and *Gallibacterium anatis*, avian leukosis virus and sicinivirus.

### 3.2. Genetic Relationships of the Tanzanian Strains with Other AAstVs

**Figure 1:**
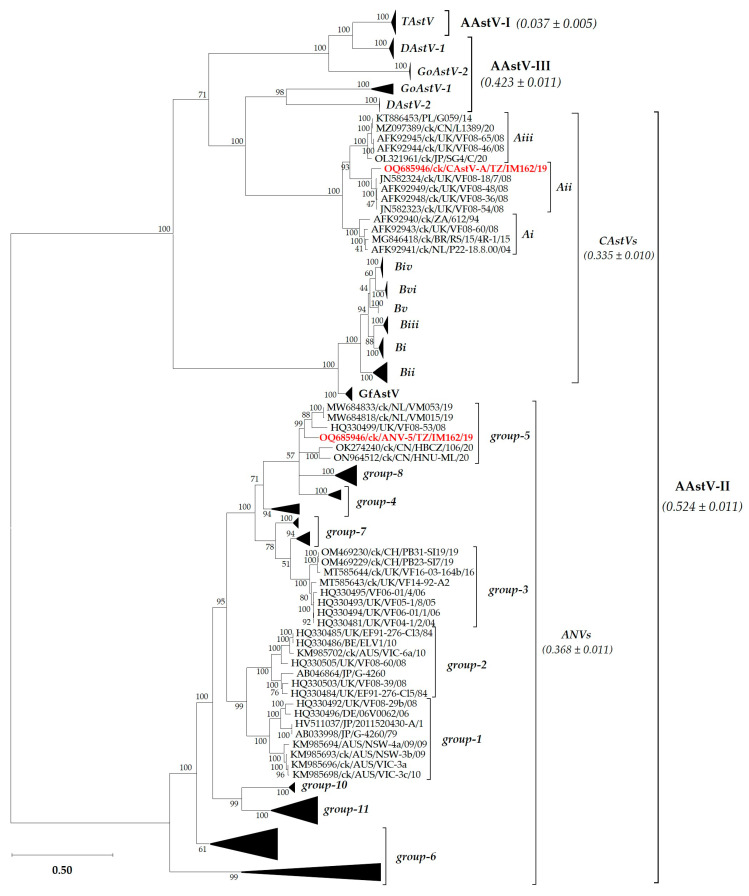
Relationship of the Tanzanian ANV and CAstV strains (highlighted in bold red font) with other AAstVs based on the complete capsid protein (CP) aa sequences. The mean genetic distances and standard deviation (±) are indicated in brackets for each group. Details of the taxa in the condensed subtrees are shown in Figure S1. The final data set involved 147 sequences and a total of 577 positions. Genogroups are named as explained in the text. Sequence names include GenBank accession numbers, abbreviated host avian species, and country/strain/strain/year of isolation. Abbreviations: AAstV, *Avastrovirus*; ANV, avian nephritis virus; CAstV, chicken astrovirus; DAstV, duck astrovirus; GfAstV, guinea fowl astrovirus; GoAstV, goose astrovirus; TAstV, turkey astrovirus.

The lengths of the gene coding regions of the Tanzanian strains are consistent with other AAtstVs that have full-length genome sequences available in GenBank (Table 1). Based on the complete CP aa sequences, CAstV and ANV phylogenetically group with, but are distinct from, European CAstV-Aii and ANV-5 viruses, respectively (Figure 1; see Figure S1 for details of the taxa in the condensed subtrees). The two Tanzanian AAstVs identified in the current study have been named as ck/ANV-5/TZ/IM162/19 and ck/CAstV-A/TZ/IM162/19 and are hereafter abbreviated as ANV-5/IM162/19 and CAstV-A/IM162/19, respectively.

The clustering of the Tanzanian strains observed in the CP tree topology was similar to the phylogenetic trees based on the sequences of the full-length genomes, nsP and RdRp (Figure S2).

The full-length genome and the nsP sequences of the Tanzanian ANV-5/IM162/19 are most similar to those of a Brazilian ANV-8 strain RS/6R/15 with nt identities of 82.72% and 86.22%, respectively. However, the CP sequences are most similar to Dutch and Chinese ANV-5 viruses with nt identities in the range of 72.2–76.68% (Table 2). The CP showed the lowest nt identities to pigeon ANV-6 viruses (42.44–46.20%) and European ANV-1 and ANV-2 viruses (56.68–58.20%). The RdRp showed the highest nt identities amongst the chicken ANVs (87–91%).

Full-length genome sequence of CAstV-A/IM162/19 is most similar (82.23% nt identity) to the Polish strain PL/G059/2014, the only subgroup A virus with a full-length genome available in GenBank (Table 3). Using the CP sequence, the Tanzanian CAstV strain is most similar to the British Aii viruses (~78% nt identity) compared to identities with Ai (69–71%) and Aiii (71.7%) viruses. The nsP and RdRp of CAstV-A/IM162/19 are most similar to the Malaysian Bv strain MY/UPM1019/18 and Swiss Bvi strain CH/PB41-SI14/19 with nt identities of about 90% and 95%, respectively.

### 3.3. Genome Sequence Analyses of Tanzanian AAstVs

#### 3.3.1. Genomic Characteristics of Strain ANV-5/IM162/19

**Figure 2:**
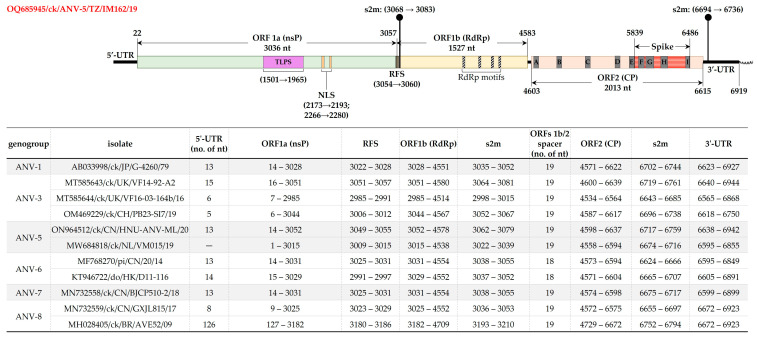
Schematic representation of the genomic organization and features of ANV-5/IM162/19 strain (upper panel) compared to other ANVs (tabulated in the lower panel). The coordinates and sizes (nt) of ORF1a, ORF1b and ORF2 (encoding nsP, RdRp and CP, respectively) are indicated. Other features shown include trypsin-like serine peptidase (TLSP), nuclear localization signal (NLS), ribosomal shift sequence (RFS), stem-loop II-like motif (s2m), 5′-/3′-unstranalated regions (UTR), and nine of the variable regions (A–I) that have been reported in ORF2 of ANVs and other AstVs [5,13,17,31,35,50]. Sequence names include GenBank accession numbers, abbreviated host avian species, and country/strain/strain/year of isolation.

Figure 2 illustrates ANV-5/IM162/19 genome organization and features compared to other ANVs (based on available genomic data [5,13,17,31,35,50]). Amongst the conserved motifs include sequence CCGAA at the beginning of the 5′-UTR (nt position 9–13), a ribosomal frame shift sequence (RFS; nt position ^3054^ AAAAAAC ^3060^) located in the overlapping region of ORF1a (3036 nt in length) and ORF1b (1527 nt in length), a trypsin-like serine peptidase (TLSP) in the middle of ORF1a (nt position 1501–1965), a bipartite nuclear localization signal (NLS) at nt positions 2173–2193 (aa residues ^725^ K**R**KGKTK ^731^; underlined arginine [R] residue varies from other strains used in the analysis, which contain lysine [K]) and an nt position 2266–2280 (aa residues ^756^ TEEEY ^760^), and a stem-loop II-like motif (s2m; nt position ^3068^ GCCCCCUUCGGGGGGC ^3083^). The coding region of ORF1a contains five predicted transmembrane domains (at nt positions 892–948, 967–1047, 1063–1137, 1171–1233 and 1267–1329). ORF1b contains the conserved RdRp motifs at positions ^234^ DWTRFD ^239^, ^295^ GNPSG ^299^, ^345^ YGDD ^348^, ^373^ FGMWVK ^378^ (Figure 2). Consistent with chicken ANVs, the ORF2 (2013 nt in length) of ANV-5/IM162/19 is separated from ORF1b by a 19 nt spacer; the genome terminates with a 3′-UTR (304 nt in length; position 6616–6919) that contains an s2m motif (position 6694–6736; except in the pigeon ANV-6 viruses with an 18 nt spacer).

**Figure 3:**
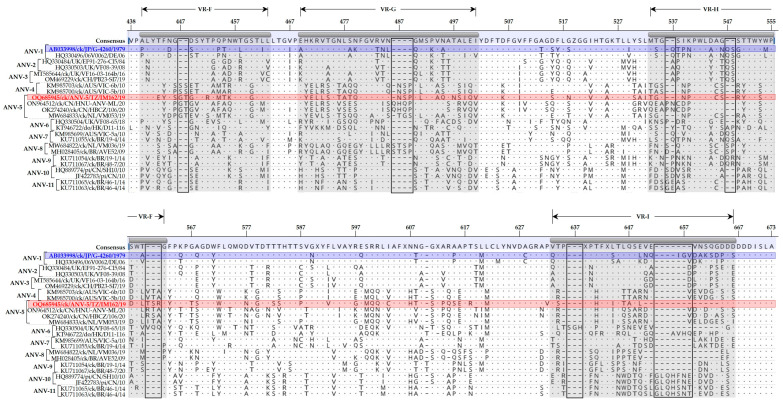
Variations in the aa residues of the spike (P2) region of the CP sequence of ANV-5/IM162/19 strain (highlighted in red font) compared to representatives of ANV genogroups 1-11 (ANV-1 to ANV-11), shown in square bracket on the left side of the alignment). The aa positions relative to the first (methionine) residue of the protein-coding CP gene are indicated at the top of the consensus (aligned) sequence. The 1979 Japanese ANV-1 strain G-4260 (highlighted in blue), which has been reported to be widely spread in Japanese and European commercial farms [13], is used as reference in the alignment, and four of the nine variable regions (VR-F to VR-I) in the P2 region of the CP sequence are highlighted (shaded in grey color). Identical and missing aa residues are indicated by dots (.) and dashes (-), respectively. Sequence names include GenBank accession numbers, abbreviated host avian species, and country/strain/strain/year of isolation.

Alignment of the coding regions of the CP sequences revealed numerous aa variations throughout the protein sequence when comparing ANV-5/IM162/19 with representative strains of ANV-1 to ANV-11. As expected, most of the aa variations are in the spike region of the CP protein [13], which are illustrated in Figure 3. The variations include substitutions in ANV-5/IM162/19 compared to other ANV-5 viruses, as well as aa insertions and deletions (indels) when comparing ANV-5 viruses to viruses belonging to other ANV subgroups.

#### 3.3.2. Genomic Characteristics of Strain CAstV-A/IM162/19

**Figure 4:**
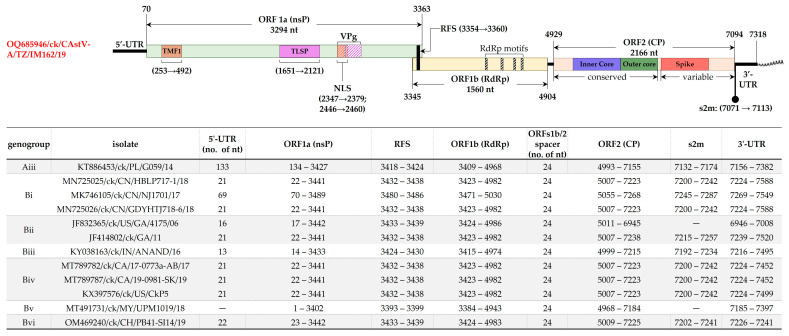
Schematic representation of the genomic organization and features of CAstV-A/IM162/19 (upper panel) compared to other CAstVs (tabulated in the lower panel). The coordinates and size (nt) of ORF1a, ORF1b and ORF2 (encoding nsP, RdRp and CP, respectively) are indicated. Other features shown include TATA element modulatory factor-1 binding domain (TMF1), trypsin-like serine peptidase (TLSP), nuclear localization signal (NLS), ribosomal shift sequence (RFS), stem-loop II-like motif (s2m), RdRp motifs, 5′-/3′-unstranalated regions (UTR), and viral genome-linked protein (VPg). The structural features are derived from available genomic datasets on AstVs [5,13,17,31,35,50]. Sequence names include GenBank accession numbers, abbreviated host avian species, and country/strain/strain/year of isolation.

Genome architecture of CAstV-A/IM162/19 is typical of CAstVs with ORF1a (3294 nt in length) and ORF1b (1560 nt in length) overlapping with a 19 nt linker that harbors RFS (position ^3354^ AAAAAAC ^3360^), and ORF2 (2166 nt in length) separated from ORF1b by a 24 nt spacer that contains the AAstV pentamer located at position ^4914^ CCGA ^4918^ (Figure 4). Based on the genome sequence data currently available in the GenBank, the 5′-UTR of CAstVs remain poorly described; there is no consensus on the lengths of this region. It is therefore possible that the 5′-UTR of the Tanzanian CAstV-A/IM162/19 reported here could be incomplete, which we are currently investigating. The three ORFs are flanked by a 5′-UTR and 3′-UTR of 69 nt and 224 nt in length, respectively; the 43 nt long s2m motif extends from the last 23 nt of ORF2 to the 3′-UTR (position 7071–7113). The coding region ORF1a has six predicted transmembrane domains (at nt positions 601–663, 1069–1125, 1183–1251, 1270–1329, 1363–1434 and 1438–1500) and NLS at nt position 2278–2310 (aa residues ^760^ KKKGKTKRTAR ^770^). Consistent with other CAstVs [17], ORF1b of CAstV-A/IM162/19 contains the RdRp motifs positioned at ^268^ DWTRFD ^273^, ^330^ GNPSG ^334^, ^380^ YGDD ^383^, ^408^ FGMWVK ^413^ (Figure 4).

**Figure 5:**
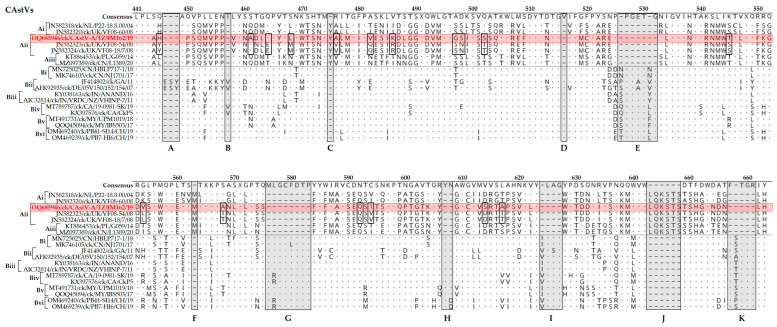
Variations in the aa residues of the spike (P2) region of the CP sequence of the Tanzanian CAstV-A/IM162/19 strain (highlighted in red) compared to representatives of CAstV subgroups Ai-iii and Bi-Bvi (shown in square bracket on the left side of the alignment). The aa positions relative to the first (methionine) residue of the protein-coding CP gene are indicated at the top of the consensus (aligned) sequence. Dots (.) indicate identical aa residues and dashes (-) indicate missing residues in shaded boxes (labeled A–K). The aa variations between the Tanzanian strain and CAstV-Aii strains from the UK are indicated by open boxes. Sequence names include GenBank accession numbers, abbreviated host avian species of origin, two-letter country abbreviation, strain/strain and year of isolation.

Heterogeneity in the spike region of the CP (the ORF2 product) was assessed by aligning translated aa sequences of representative strains of CAstV-A (subgroups i-Aiii and CAstV (subgroups Bi to Bvi). The alignment segregated CAstV-A from CAstV-B viruses, with 11 indel regions between the two groups (arbitrarily named A to K in the figure), and with 17 aa substitutions when comparing CAstV-A/IM162/19 and other analyzed CAstV-Aii viruses (Figure 5). CAstV-A and B viruses shared only pockets of 1–3 identical aa residues. Overall, the spike region of CAstV-A viruses is more heterogenous than CAstV-B viruses which share 77.0% and 82.2% aa identical sites, respectively.

### 3.4. Analyses of Recombination Events

**Figure 6:**
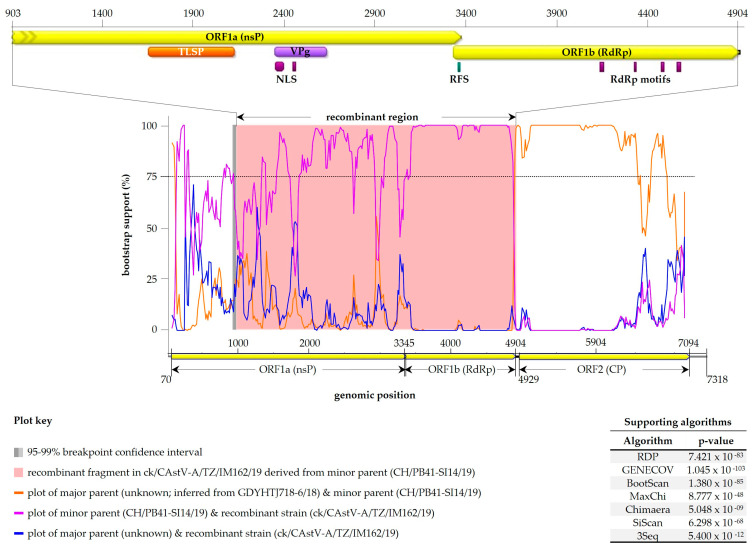
Plot of recombinant fragment identified in the Tanzanian CAstV-A/IM162/19 strain. The top panel shows recombinant fragment genomic position in CAstV-A/IM162/19, which corresponds to the pink area bounded by recombination breakpoints (bottom panel). The recombination signal was supported by seven RDP4 methods (table at bottom right), with a Swiss strain CH/PB41-SI14/19 (GenBank accession: OM469240) as the “minor parent”—strain most identical to the recombinant fragment in CAstV-A/IM162/19 (in this case at 92.6% nt identity). The “major parent” (i.e., strain most identical to CAstV-A/IM162/19 in the genomic region surrounding the recombinant breakpoints) is unknown, but RDP4 inferred it to be a Chinese CAstV-Bvi strain GDYHTJ718-6/18 (GenBank accession: MN725026). The vertical dotted line indicates bootstrap cutoff (75%) used to determine the significance of the breakpoints. Domain features (described in Figure 4) in the recombinant fragment are shown in the top panel.

Because of the diversity observed in our analyses and reports of their occurrence in AAstVs in poultry [28], we assessed the possible occurrence of recombination events in the Tanzanian AAstVs using the RDP4 suit, which involved the 78 full-length genome sequences used for the phylogenetic analyses shown in Figure S1. The analyses detected a recombination signal in strain CAstV-A/IM162/19, with the recombinant fragment covering 75% of the C-terminal region of ORF1a (2461 nt), the entire ORF1b (1560 nt) and 16 out of the 24 nt in the ORF1b/ORF2 spacer region (Figure 6).

The predicted minor parent strain, which is defined here as the strain most identical to the recombinant fragment in CAstV-A/IM162/19, is a Swiss CAstV-Bvi strain CH/PB41-SI14/19 (GenBank accession: OM469240); the 4018 nt recombinant fragment in the Tanzanian strain is 92.6% identical to the homologous region in the Swiss virus. The Swiss virus was detected from an RSS-affected broiler flock [51].The major parental strain, which is defined here as the strain with the genomic region surrounding the recombinant breakpoint most similar to the recombinant strain (i.e., CAstV-A/IM162/19), could not be identified amongst the analyzed viruses. However, RDP4 inferred the major parent to be also a CAstV-Bi strain; the closest match is a Chinese strain (GDYHTJ718-6/18; GenBank accession: MN725026), which was reported as a novel AAstV from a sero-positive chicken flock [52]. Our analyses did not detect recombinant signals in the two parental strains.

A recombinant event signal was also detected in ORF1a (647–1439 nt region, which contains the viroporin domain) of ANV-5/IM162/19, with a British genogroup ANV-3 strain UK/VF14-92-A2 (GenBank accession: MT585643) as the major parent and an unknown minor parental strain. Although this recombination event signal was supported by six out of the nine RDP4 detection algorithms, it was deemed a misidentification because the beginning of the breakpoint was uncertain and the parental strain UK/VF14-92-A2 was suggested as the likely actual recombinant.

## 4. Discussion

The AAstVs are among the least-studied avian enteric RNA viruses. Recent advances in the use of NGS technologies have however led to the discovery of many novel AAstVs, complicating classification because the current species are demarcated based on their genetic distances within the CP aa sequences, and the phylogenetic clustering of the viruses does not necessarily correspond to the classification. The vast majority of the AAstV genomic data available in the databases are partial sequences of ORF2 (capsid gene), and fewer of ORF1b (RdRp gene). Furthermore, only a few complete genome sequences of ANVs and CAstVs are available, most of which are ANV-3/-6/-8 and CAstV-Biv/vi strains. Several of the AAstV strains in the databases have little or no molecular characterization and remain unclassified. In this study, we identified and characterized the full-length genome sequences of two AAstVs assembled *de novo* into single contigs, from the NGS of a cloacal swab obtained from a backyard chicken originating from rural Tanzania and sold at an urban LBM in the Iringa region of the country.

Although identified in a single bird, the two Tanzanian strains belong to two different genogroups of AAstVs, which is not surprising because strains from different genogroups have been reported to simultaneously infect a single avian species [35,53,54]. There is also evidence that coinfection of poultry with multiple enteric viruses could result in an increase in the severity of enteritis and the risk to infected animals of secondary infections with opportunistic microbial pathogens [55]. The chicken from which the cloacal sample was obtained contained detectable levels of RNAs of bacterial species that are associated with poultry diseases as opportunistic pathogens, which included *E. cecorum*, *E. faecium*, *Avibacterium* and *Gallibacterium anatis*. We also detected RNAs of an endogenous retrovirus (avian leukosis virus subgroup; ALV-E) and a picornavirus (sicinivirus type A; SiV-A). However, because there were no recorded metadata about any overt clinical signs of disease in the sampled birds, we could not associate the Tanzanian AAstV strains to pathologies in chickens.

The full-length genome sequences of ANV-5/IM162/19 (6919 nt) and CAstV-A/IM162/19 (7318 nt) and their architecture (5′-UTR-ORF1a-ORF1b-ORF2-3′-UTR) are consistent with other AAstVs [35]. The observed variations in sequence lengths of the full-length genomes and individual ORFs amongst the strains used in our analyses are not unexpected for AAstVs; such variations are largely attributable to viral molecular evolution resulting from factors such as recombination events, indels, and the ability of these viruses to be transmitted vertically (via eggs) and across different avian species, as well as other unexplored biological and ecological factors [28,35,56,57,58,59,60]. Recombination events have intrinsic potential to complicate the classification of RNA viruses [60], and even more so in the case of AAstVs challenged with insufficient full-genomic data. Indels are more common in the variable genomic regions of RNA viruses (specifically in the spike region of the capsid protein in the case of AAstVs) than in conserved regions (ORF1a and ORF1b, which encode the nsP and RdRp of AAstVs, respectively), where recombination events occur with higher frequencies between genetically related species [35]. There were higher identities amongst the analyzed nsP and RdRp sequences compared to the much lower and wide-ranging identities amongst the CP sequences. Numerous aa variations were also observed in the spike region, which is to be expected because it is the most variable genomic region of AAstVs [11,12,20]. Our analyses of the CP sequences show that the three subgroups of CAstV-A (i–iii) cluster distinctly, unlike the six subgroups of CAstV-B (i–vi) which cluster with guinea-fowl AAstVs (GfAstVs). Furthermore, based on the CP sequences, the Chinese AAstV-III duck type 2 (DAstV-2) and goose type 1 (GoAstV-1) viruses phylogenetically cluster with the CAstV viruses separate from DAstV-1 and GoAstV-2. Overall, the phylogenetic clustering of the Tanzanian AAstVs was consistent based on the full genome and all three ORF sequences, but they appear to be distinct from other strains through their respective ANV and CAstV groups.

The recombination event detected in the nsP/RdRp genomic region of CAstV-A/IM162/19 underscores the potential of recombination events complicating the classification of AAstVs. The proposed parental strains of the recombinant fragment in CAstV-A/IM162/19 (CAstV-Aii) are CAstV-B viruses from different subgroups (i.e., Bi and Bvi), which is interesting because, as stated above, recombination events mostly occur between closely related viral species. However, it should be noted that most of the available CAstV full-length genome sequence data are of Eurasian and Canadian genogroup B viruses, creating the possibility that the real parental strains of the Tanzanian recombinant strain are yet to be identified. Many recombinants have been reported from Canada [28]. The classification of AAstVs has hitherto included partial CP sequences (as opposed to using complete protein coding sequences); our data indicate that expansion of the phylogenetic analyses, and the classification of these viruses using the sequences of the full-length genome and all three ORFs, should be considered.

An important aspect to highlight is that the European AAstVs to which the Tanzanian strains are most closely related (phylogenetically and nt/aa identities) have been implicated in gastrointestinal diseases and hatchability problems in commercial poultry production [29]. Our analyses have shown high levels of heterogeneity in the CP of the Tanzanian strains compared to the European viruses. However, the implications of the genetic differences between the Tanzanian and the European viruses in terms of pathology remain unknown. Furthermore, viral pathogenicity can be a subject of myriads of other factors such as viral loads, age, breed and immune status of the host birds, and co-infecting pathogens. Nevertheless, because the CP contains the vast majority of the viral epitopes that are exposed to and interact with the host’s immune system, the numerous genetic aa variations observed in this region amongst the AAstVs are of obvious significance in future epidemiological investigations into AstVs, as well as in the development of diagnostic tools and vaccines in efforts to control these viruses. With the difficulties currently associated with the growth of AAstVs in cell culture, the nontargeted approach is an attractive alternative to PCR/Sanger sequencing in molecular studies of these viruses as demonstrated by the data and results of the current study.

## 5. Conclusions

We have presented full-length genome sequences and the characterization of CAstV and ANV strains assembled *de novo* from the NGS of a cloacal swab from a backyard chicken in rural Tanzania, which is a first in the East and Central African regions. Based on comparative sequence analyses and phylogenetics using the currently available genomic data on AAstVs, the two Tanzanian strains belong to two different genogroups and are most closely related to Eurasian viruses, but with numerous aa variations in the spike region of the capsid protein that harbors the majority of the viral epitopes. We have also demonstrated that the Tanzanian CAstV strain has a CAstV-Aii backbone with a potential recombinant fragment in the conserved genomic region (nsP/RdRp) derived from CAstV-B parental viruses. These data provide a valuable contribution to the repertoire of genomic datasets of AAstVs and hint at the need for revisiting the current ICTV taxonomic classification of members of the *Astrovirus* genus. Furthermore, the observed heterogeneity of the CP should inform on the future development of diagnostics and vaccines for the management of AAstVs.

## Figures and Tables

**Table 1:** Comparison of the gene coding regions of the Tanzanian strains (in bold text) to other AAstVs. The genome lengths exclude the 5′-/3′-UTRs and poly(A) tails.

Group	Subgroup	Origin (GenBank Accession Numbers)	length (Nucleotides)			
			Genome	ORF1a	ORF1b	ORF2
ANV	1	Japan (HV511037; AB033998)	6609	3018	1542	2145
	3	Switzerland (OM469230; OM469229); UK (MT585644M; MT585643)	5847–6624	2277–3045	1452–1578	2124–2424
	5	**Tanzania (OQ685945)**	**6594**	**3042**	**1452**	**2106**
		Netherlands (MW684818; MW684833); China (MW784086; ON964512)	6543–6776	2967–3045	1452–1587	2130–2142
	6	China (MW784094; MF768270); Hong Kong (KT946723; KT946722; KT946724; KT946725)	6575–6599	3015–3021	1452–1539	2019–2043
	7	China (MN732558)	6585	3021	1452	2118
	8	Netherlands (MW684822); Brazil (MH028405); China (MN732559)	6513–6567	2967–3063	1452–1539	2019–2100
CAstV	Aii	**Tanzania (OQ685946)**	**7025**	**3294**	**1560**	**2166**
	Aiii	Poland (KT886453)	7148	3420	1560	2163
	Bi	China (MK746105; MN807051; MN725026; MN725025)	7199–7202	3420	1560	2124–2217
	Bii	USA (JF832365; JF414802); Switzerland (OM469245)	6929–7217	3420–3426	1560–1563	1935–2232
	Biv	USA (KX397576); Canada (MT789774; MT789777; MT789778; MT789780; MT789782; MT789785; MT789787)	7202–7219	3420–3423	1560	2217
	Bv	Malaysia (MT491731)	7090	3294	1560	2217
	Bvi	Netherlands (MW684830); Switzerland (OM469239; OM469240; OM469242)	6919–7216	3123–3420	1560	2217

**Table 2:** Comparative identities of the Tanzanian strain ANV-5/IM162/19 with other ANVs. Highest nucleotide (nt; and corresponding aa in brackets) identities are in bold and underlined and the lowest identities are italicized. Strains without full-length genome sequences are indicated with a dash (–) in their corresponding ORF1a and OFR1b.

Subgroup	Virus *	Pairwise Comparative Identities (%)			
		Genome (nt)	ORF1a nt (aa)	ORF1b nt (aa)	ORF2 (nt (aa)
ANV-1	AB033998/JP/G-4260/79	75.12	78.06 (80.16)	87.26 (94.82)	58.11 (56.69)
	HQ330496/DE/06V0062/06	–	–	–	57.51 (57.58)
	KM985694/AUS/NSW-4a/09/09	–	–	–	58.20 (56.73)
ANV-2	HQ330486/Belgian-ELV1	–	–	–	56.68 (56.87)
	HQ330503/UK/VF08-39/08	–	–	–	57.30 (56.58)
	HQ330484/UK/EF91-276-Cl5/84	–	–	–	57.64 (56.15)
ANV-3	MT585643/ck/UK/VF14-92-A2	78.39	78.93 (81.86)	**90.98** (94.20)	65.36 (67.63)
	MT585644/ck/UK/VF16-03-164b/16	77.90	78.73 (82.16)	90.08 (94.41)	65.21 (66.91)
	OM469229/ck/CH/PB23-SI7/19	78.37	80.64 (82.23)	89.88 (95.24)	65.16 (66.91)
ANV-5	ON964512/ck/CN/HNU-ANV-ML/20	**81.97**	81.18 (82.69)	90.43 (94.62)	72.73 (78.71)
	MW684818/ck/NL/VM015/19	**82.17**	79.83 (82.95)	89.94 (**95.45**)	**77.22 (85.25)**
	MW684833/ck/NL/VM053/19	80.70	76.67 (78.10)	90.15 (95.24)	**77.22 (85.25)**
	MW784086/ck/CN/232-76811/22	77.49	79.42 (80.48)	90.43 (94.82)	63.70 (64.84)
	OK274240/ck/CN/HBCZ/106/20	–	–	–	76.48 (82.40)
ANV-6	MF768270/pigeon/CN/20/14	*63.88*	*69.07 (69.78)*	*75.96 (84.27)*	*42.44 (37.57)*
	FR727147/pigeon/NO/594-9/03	–	–	–	43.03 (38.40)
	FR727148/pigeon/NO/603-5/03	–	–	–	*43.28 (38.86)*
	FR727149/ pigeon/NO/06/15660-1/05	–	–	–	*46.20 (43.47)*
ANV-7	MN732558/ck/CN/BJCP510-2/18	76.70	77.34 (79.76)	89.26 (94.41)	64.91 (66.03)
ANV-8	MH028405/ck/BR/AVE52/09	78.78	77.34 (80.00)	90.84 (94.62)	69.33 (72.78)
	MN732559/ck/CN/GXJL815/17	80.39	79.93 (82.37)	89.33 (95.24)	71.09 (74.70)
	MW684822/ck/NL/VM036/19	79.99	80.36 (83.85)	90.56 (94.62)	68.94 (73.48)
	MG846415/ck/BR/RS/6R/15	**82.72**	**86.22 (91.02)**	90.01 (94.82)	69.73 (74.26)

* virus names include GenBank accession numbers, abbreviated host avian species of origin, two-letter country abbreviation, strain/strain and year of isolation.

**Table 3:** Comparative identities of the Tanzanian strain CAstV-A/IM162/19 with other CAstVs. Highest nucleotide (nt; and corresponding aa in brackets) identities are in bold and underlined and the lowest identities are italicized. Strains without full-length genome sequences are indicated with a dash (–) in their corresponding ORF1a and OFR1b.

Subgroup	Virus *	Pairwise Comparative Identities (%)			
		Genome (nt)	ORF1a nt (aa)	ORF1b nt (aa)	ORF2 nt (aa)
Ai	MG846418/ck/BR/RS/15/4R-1/15	–	–	–	71.27 (76.38)
	AFK92941/ck/NL/P22-18.8.00/04	–	–	–	69.71 (76.80)
	AFK92943/ck/UK/VF08-60/08	–	–	–	70.63 (77.49)
	AFK92940/ck/ZA/612/94	–	–	–	70.03 (76.93)
Aii	AFK92948/ck/UK/VF08-36/08	–	–	–	77.55 (90.04)
	AFK92949/ck/UK/VF08-48/08	–	–	–	77.59 (90.18)
	JN582324/ck/UK/VF08-18/7/08	–	–	–	**78.19** (90.46)
	JN582323/ck/UK/VF08-54/08	–	–	–	78.05 (**90.73**)
Aiii	KT886453/ck/PL/G059/14	**82.23**	86.92 (94.07)	87.12 (94.03)	71.69 (79.94)
	MZ097389/ck/CN/L1389/20	–	–	–	71.69 (79.94)
	AFK92945/ck/UK/VF08-65/08	–	–	–	71.69 (79.67)
	AFK92944/ck/UK/VF08-46/2008	–	–	–	71.69 (79.67)
Bi	MN725025/CN/HBLP717-1/18	*66.69*	*75.96 (86.78)*	*78.08 (85.93)*	47.26 (38.74)
	MK746105/ck/CN/NJ1701/17	*66.83*	*76.23 (86.87)*	*78.21 (86.32)*	47.17 (38.92)
	MN725026/ck/CN/GDYHTJ718-6/18	*66.63*	*76.2 (86.33)*	*77.56 (85.93)*	47.19 (38.74)
	MW446478/tk/NG/VF18-18/64/17	–	–	–	46.94 (39.13)
Bii	JF832365/ck/USA/GA/4175/06	74.4	87.27 (93.36)	92.45 (85.96)	*42.47 (32.35)*
	JF414802/ck/GA2011	75.74	88.4 (94.44)	94.29 (97.3)	46.35 (39.48)
	OM469245/ck/PB23-SI7/CH/19	–	–	–	46.48 (39.08)
	AFK92935/ck/DE/05V150/152/154/07	–	–	–	45.88 (39.35)
Biii	KY038163/ck/IN/ANAND/16	74.91	87.52 (93.89)	91.73 (96.15)	47.68 (39.66)
	JX945853/ck/IN/PDRC/200/11	–	–	–	47.76 (39.39)
	KC618323/ck/IN/PDRC/447/12	–	–	–	47.04 (38.68)
	AIC32814/ck/IN/VRDC/NZ/VHINP-7/11	–	–	–	47.50 (39.53)
Biv	MT789782/ck/CA/17-0773a-AB/17	75.23	87.61 (92.43)	93.65 (97.5)	45.95 (39.53)
	MT789787/ck/CA/19-0981-SK/19	74.44	87.43 (94.07)	90.58 (96.34)	46.04 (39.66)
	KX397576/ck/USA/CkP5	74.8	87.74 (94.62)	92.05 (96.72)	45.82 (39.79)
Bv	MT491731/ck/MY/UPM1019/18	76.52	**89.74** (**95.08**)	95.06 (98.07)	46.75 (39.92)
	QOQ45094/ck/MY/IBS503/17	–	–	–	46.75 (39.92)
	QOQ45100/ck/MY/IBS543/17	–	–	–	46.75 (39.92)
Bvi	OM469240/ck/CH/PB41-SI14/19	76.51	89.5 (94.99)	**95.90** (**98.46**)	46.75 (39.92)
	OM469242/ck/CH/PB15-HI11/19	–	–	–	45.95 (40.05)
	OM469239/ck/PB7-HI6/CH/19	–	–	–	46.04 (40.05)
	MW684830/ck/NL/VM046/19	–	–	–	45.91 (40.18)

* virus names include GenBank accession numbers, abbreviated host avian species of origin, two-letter country abbreviation, strain/strain and year of isolation.

## Data Availability

The full-length genome nucleotide sequence data of the Tanzanian astroviruses reported in this paper have been submitted to the GenBank database and have been assigned GenBank accession OQ685945 (strain ck/ANV-5/TZ/IM162/19) and OQ685946 (strain ck/CAstV-A/TZ/IM162/19). The raw data deposited in the SRA are under the accession numbers SRR23899252, BioSample number SAMN33770568, and BioProject number PRJNA945007.

## Supplementary Materials

The following supporting information can be downloaded at: https://www.mdpi.com/article/10.3390/v15061247/s1, Figure S1: Relationship of the Tanzanian ANV-5/IM162/19 and CAstV-A/IM162/19 strains (in red bold red) with other AAstVs based on the complete capsid protein (CP) aa sequences. Details of the condensed taxa subtrees in panel A are shown in details panel B and vice versa. Mean genetic distances and standard deviation (±) are indicated in brackets for each group. Reconstruction of the phylogenetic tree using MEGA and naming of the AAstV genogroups were performed as explained in the text with the final dataset involving 147 sequences and 577 positions. Sequence names include GenBank accession numbers, abbreviated host avian species, and country/strain/strain/year of isolation. Abbreviations: AAstV, Avastrovirus; ANV; avian nephritis virus; CAstV, chicken astrovirus; DAstV, duck astrovirus; GfAstV, guinea fowl astrovirus; GoAstV, goose astrovirus; TAstV, turkey astrovirus. Figure S2: Relationships of the Tanzanian ANV-5/IM162/19 and CAstV-A/IM162/19 strains (in red bold red) identified in the current study with AAstVs based on the nucleotide sequences of the full-length genomes and ORF1a and ORF1b sequences. Reconstruction of the phylogenetic tree using MEGA and naming of the AAstV genogroups were performed as explained in the text and involved final datasets of 78 nt sequences and 5925, 898 and 568 positions (genome, ORF1a and ORF2 sequences, respectively).
