# Supplementary material for: Genome Sequences and Characterization of Chicken Astrovirus and Avian Nephritis Virus from Tanzanian Live Bird Markets
Source: Viruses. 2023 May 25;15(6):1247. doi: 10.3390/v15061247 (PMC10302499; doi:10.3390/v15061247)
Supplement: Supplementary file 1 [file viruses-15-01247-s001.zip › viruses-2399270-supplementary.pdf]

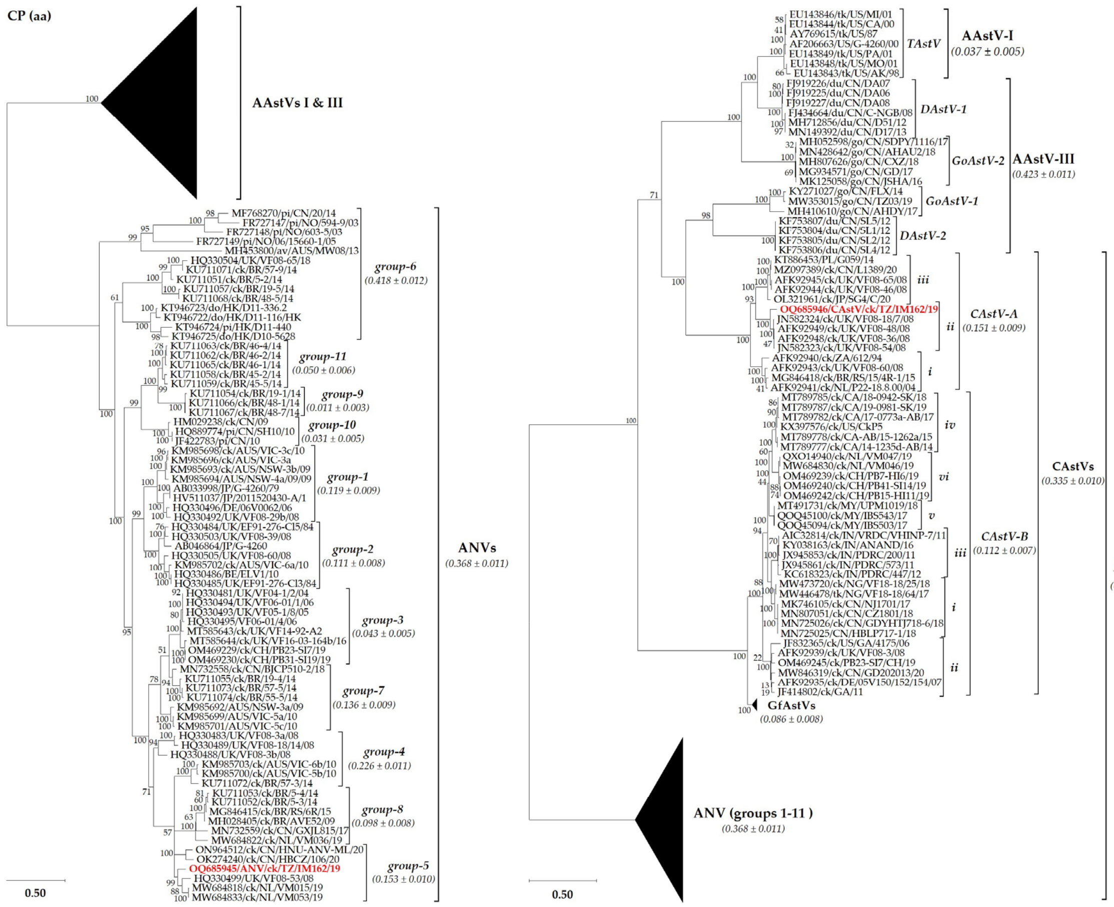

Figure S1: Relationship of the Tanzanian ANV-5/IM162/19 and CAstV-A/IM162/19 strains (in red bold red) with other AAstVs based on the complete capsid protein (CP) aa sequences. Details of the condensed taxa subtrees in panel A are shown in details panel B and vice versa. Mean genetic distances and standard deviation (±) are indicated in brackets for each group. Reconstruction of the phylogenetic tree using MEGA and naming of the AAstV genogroups were performed as explained in the text with the final dataset involving 147 sequences and 577 positions. Sequence names include GenBank accession numbers, abbreviated host avian species, and country/strain/strain/year of isolation. Abbreviations: AAstV, Avastrovirus; ANV; avian nephritis virus; CAstV, chicken astrovirus; DAstV, duck astrovirus; GfAstV, guinea fowl astrovirus; GoAstV, goose astrovirus; TAstV, turkey astrovirus.

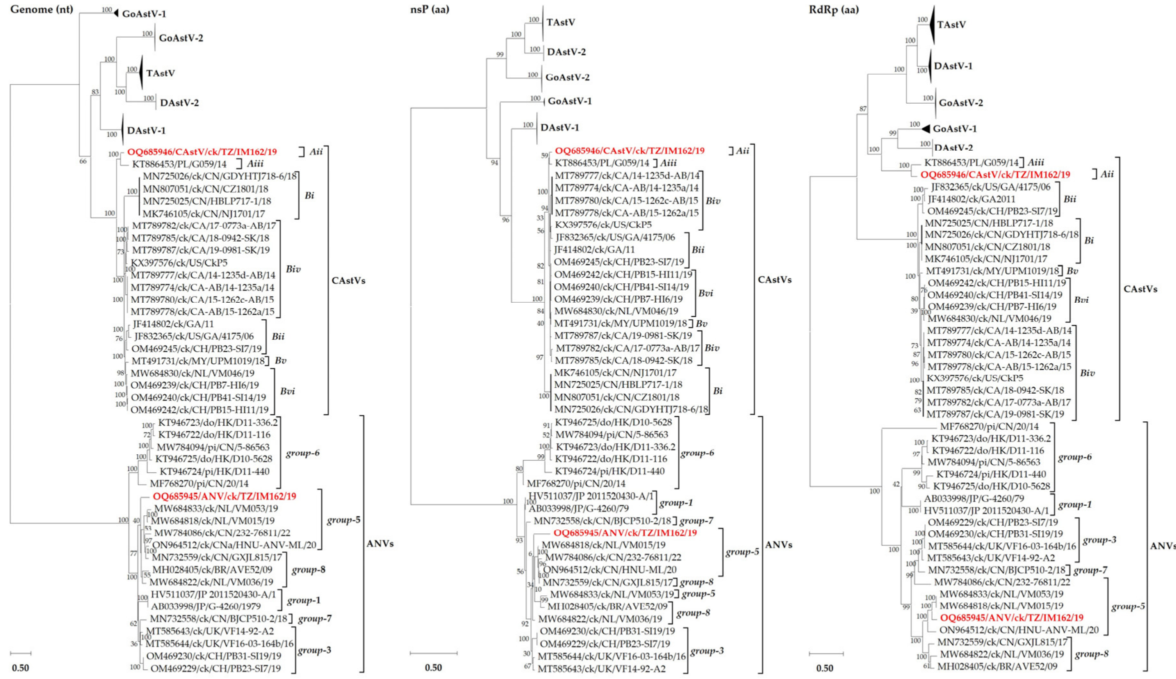

Figure S2: Relationships of the Tanzanian ANV-5/IM162/19 and CAstV-A/IM162/19 strains (in red bold red) identified in the current study with AAstVs based on the nucleotide sequences of the full-length genomes and ORF1a and ORF1b sequences. Reconstruction of the phylogenetic tree using MEGA and naming of the AAstV genogroups were performed as explained in the text and involved final datasets of 78 nt sequences and 5925, 898 and 568 positions (genome, ORF1a and ORF2 sequences, respectively).
