# Supplementary figures and images for: Genome Sequences and Characterization of Chicken Astrovirus and Avian Nephritis Virus from Tanzanian Live Bird Markets
Source: Viruses. 2023 May 25;15(6):1247. doi: 10.3390/v15061247 (PMC10302499; doi:10.3390/v15061247)

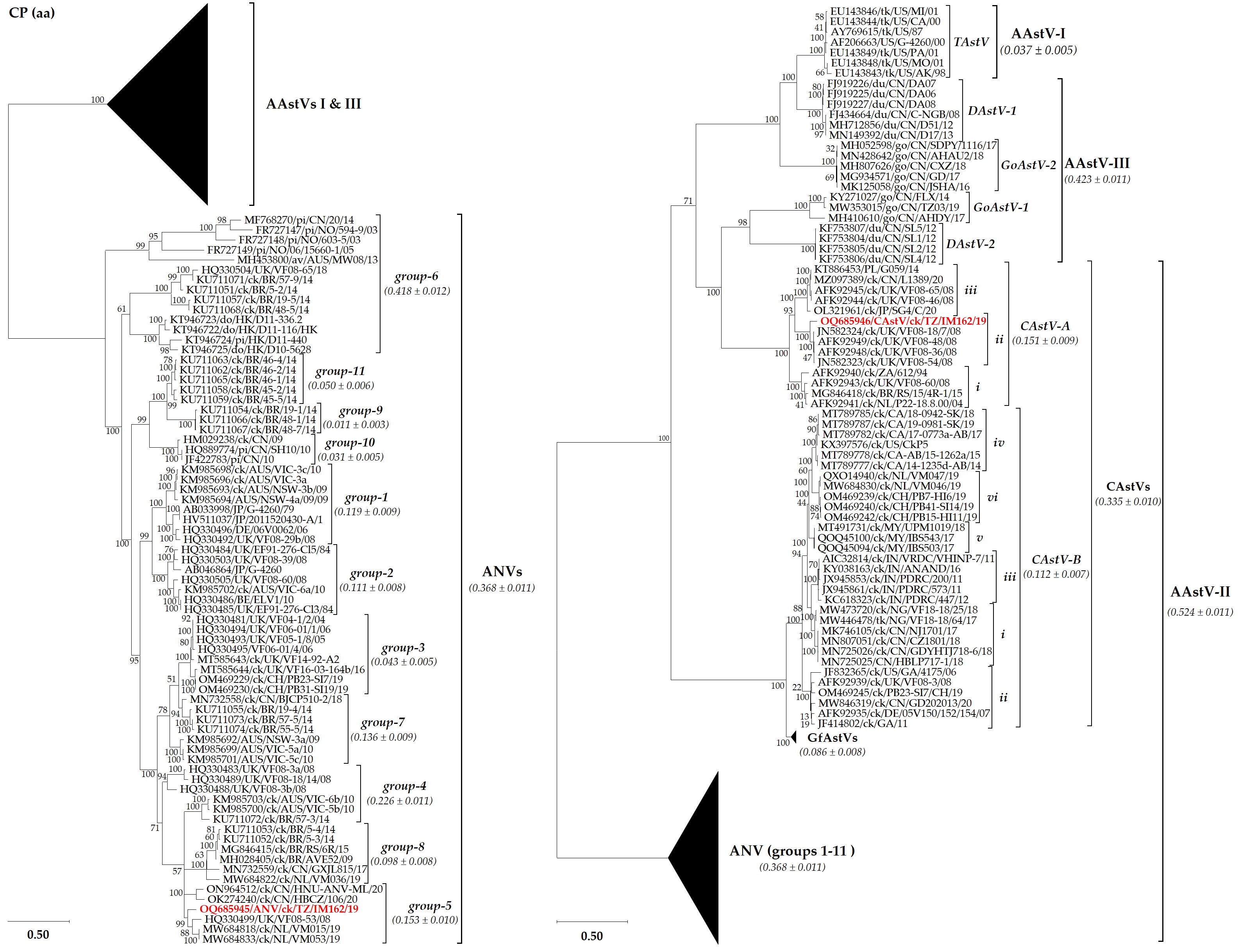

Supplement: Supplementary file 1 [file viruses-15-01247-s001.zip › SUPPLEMENTARY/FIG. S1.jpg]

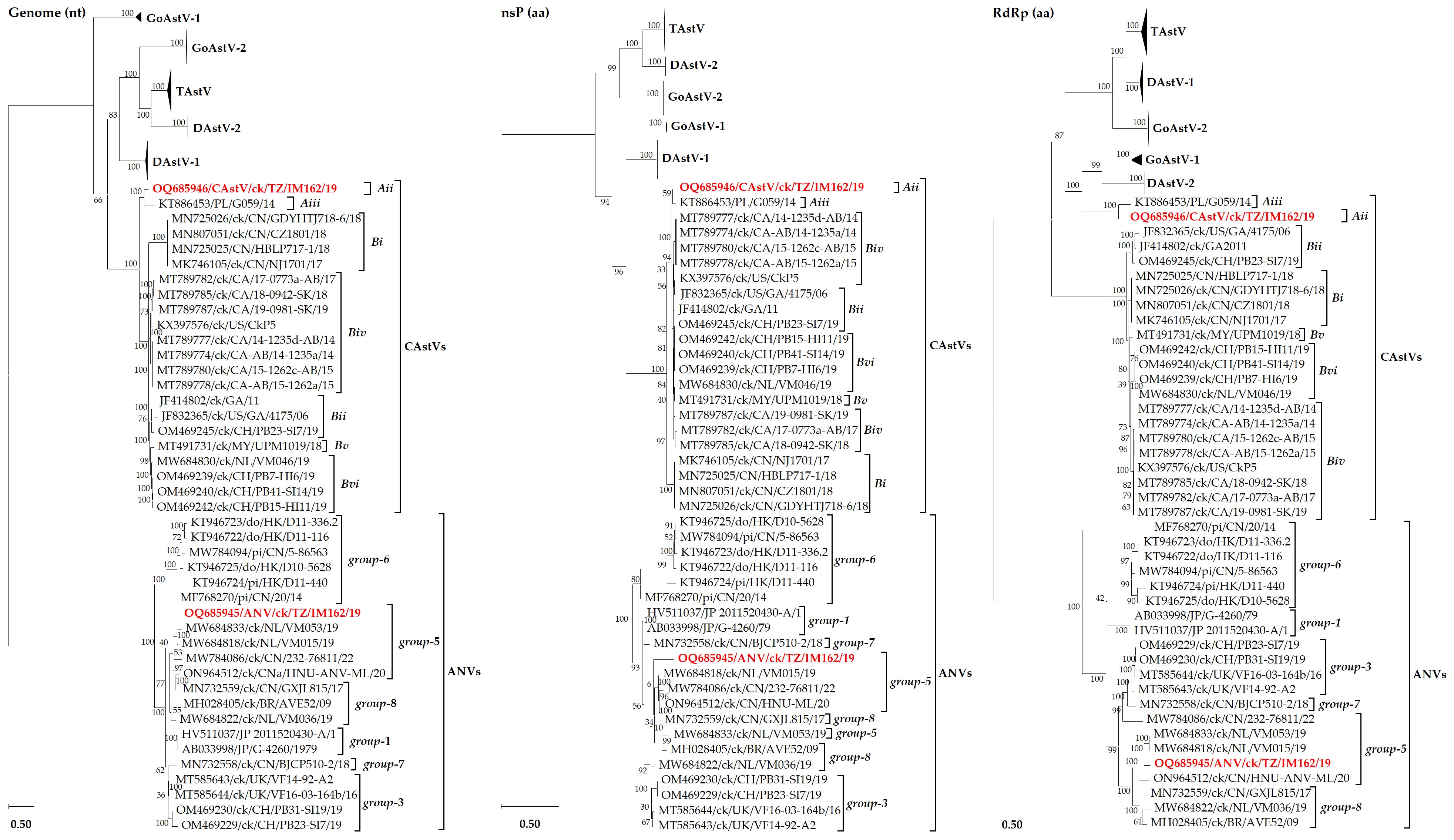

Supplement: Supplementary file 1 [file viruses-15-01247-s001.zip › SUPPLEMENTARY/FIG. S2.jpg]
